# The Causal Association Between HIV and Atrial Fibrillation: A Mendelian Randomization Study

**DOI:** 10.1155/cdr/7645834

**Published:** 2026-11-14

**Authors:** Yujia Wu, Wanqian Pan, Hang Li, Rui Han, Minyu Zhou, Xintao Li, Tingbo Jiang, Xichen Chai

**Affiliations:** ^1^ Department of Cardiology The First Affiliated Hospital of Soochow University Suzhou Jiangsu China sdfyy.cn; ^2^ Department of Cardiovascular Medicine Hainan Medical University First School of Clinical Medicine: The First Affiliated Hospital of Hainan Medical University Haikou China

**Keywords:** acquired immunodeficiency syndrome, atrial fibrillation, HIV, human immunodeficiency virus, Mendelian randomization

## Abstract

**Background:**

Human immunodeficiency virus (HIV) associated cardiovascular burden has become increasingly prominent. Previous evidence suggested an elevated risk of atrial fibrillation (AF) among HIV patients; it remains unclear whether this relationship is causal. Our study was to investigate the causal effect of HIV infection on AF.

**Methods:**

A two‐sample Mendelian randomization (MR) framework was implemented to examine causal association between HIV infection and AF. Summary data on the association of single nucleotide polymorphisms (SNPs) with HIV infection were obtained from public genome‐wide association studies. Statistical data for SNPs‐AF associations were extracted from OPENGWAS datasets. Bidirectional univariable and multivariable MR analyses were conducted to assess causal effects.

**Results:**

Univariate MR analysis revealed a suggestive causal association between HIV infection and AF risk (IVW: OR = 1.016, 95% CI: 1.004–1.029, *p* = 0.01), with no significant heterogeneity or pleiotropy. None significant effect was observed for AF on HIV risk (OR = 1.064, 95% CI: 0.839–1.350, *p* = 0.61). Multivariable MR analysis revealed an independent association between HIV infection and AF after adjusting for potential confounders (OR = 1.016, 95% CI: 1.002–1.031, *p* = 0.03).

**Conclusion:**

Our study found evidence supporting a causal association between genetically predicted HIV susceptibility and AF in individuals of European ancestry, suggesting that HIV‐related pathways may contribute to AF development.

## 1. Introduction

Atrial fibrillation (AF) is one of the most common cardiac arrhythmias and is characterized by uncoordinated atrial activation. AF is associated with increased risks of ischemic stroke, heart failure (HF), and mortality [1]. The global burden of AF is substantial and continues to rise. Estimates indicate that the prevalence of AF in the United States was 5.2 million in 2010 and is projected to increase to 12.1 million by 2030 [2]. Compared with individuals without AF, those with AF face approximately twice the risk of hospitalization and per capita medical costs [2]. Despite substantial advances in pharmacotherapy, catheter ablation, and management of conventional risk factors, long‐term AF recurrence remains common and prognosis remains suboptimal [3]. These limitations underscore the continued need to improve strategies for the prevention and management of AF.

Acquired immunodeficiency syndrome (AIDS), caused by human immunodeficiency virus (HIV) infection, is characterized by profound impairment of immune function. Its high prevalence and mortality contribute to the global disease burden. According to the latest data from UNAIDS, approximately 39.9 million people were living with HIV (PLWH) worldwide in 2023, with approximately 630,000 HIV‐related deaths reported that year [4]. Despite continued advances in HIV treatment and substantial reductions in mortality [5], over 600,000 people still died from AIDS‐related diseases in 2024. As survival improves, cardiovascular disease (CVD) has emerged as an increasingly important contributor to morbidity and mortality among PLWH. Over the past 2 decades, the HIV‐related CVD burden has tripled, resulting in approximately 2.6 million disability‐adjusted life years lost annually [6]. Among cardiovascular conditions, AF warrants particular attention because of its associations with stroke, HF, and all‐cause mortality. Emerging observational epidemiological research has suggested that PLWH may have a higher susceptibility to AF [7–10]. This association has been reported in multiple cohort studies and meta‐analyses.

However, the observed association may be confounded by factors that are independently associated with AF and differ in prevalence between PLWH and the general population. Smoking and alcohol consumption are well‐established behavioral risk factors for AF [11, 12], and both coaggregate in PLWH [13]. Obesity, hypertension, and myocardial infarction (MI) are also more prevalent in PLWH, and obesity and hypertension may additionally be influenced by antiretroviral therapy [6, 14]. All three conditions are established risk factors for AF [15–17]. Furthermore, HIV infection has been associated with accelerated biological aging even among individuals receiving effective treatment, a process that may not be fully captured by chronological age or traditional risk factors [18]. We therefore incorporated GrimAge, an epigenetic measure, to account for this dimension of biological aging. Together, these six factors capture major behavioral, metabolic, cardiovascular, and aging‐related pathways that could confound or contribute to the HIV‐AF association. Accounting for these factors within a multivariable MR framework may therefore help estimate the association between genetically predicted HIV susceptibility and AF independently of these potential pathways.

In our study, we applied both univariable and multivariable Mendelian randomization (MR) to systematically assess the potential causal relationship between genetically predicted HIV susceptibility and AF.

## 2. Method

### 2.1. Study Design

We employed a two‐stage MR analysis. First, we performed a univariable bidirectional MR analysis, which should satisfy the following three assumptions: Firstly, IV must be strongly correlated with exposure; secondly, IV is independent of any confounders; thirdly, IV affects the outcome exclusively through exposure [19].

Then we performed a multivariable MR analysis to estimate the independent causal effect of HIV on AF. We incorporated genetic instruments for multiple potentially correlated exposures into the model, such as alcohol consumption, obesity, smoking, hypertension, MI, and GrimAge. The assumptions of MVMR are consistent with those of univariable MR but are extended to the multivariable context [20]. We first incorporated each potential confounder individually, and then included all confounders simultaneously for joint adjustment. Our study design is presented in Figure 1.

**Figure 1:**
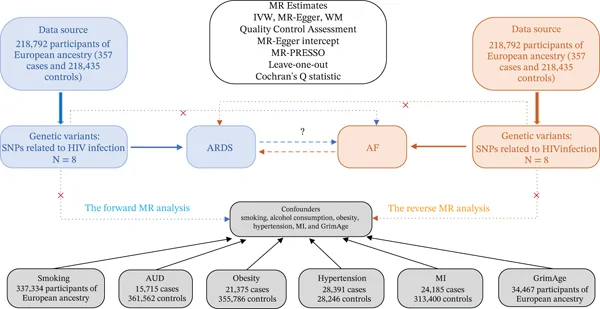
Study overview. SNP, single nucleotide polymorphism; MR, mendelian randomization; IVW, inverse‐variance weighted; WM, weighted median; MR‐PRESSO, Mendelian randomization pleiotropy residual sum and outlier; Cochran′s *Q* test, Cochran′s *Q* heterogeneity test; MI, myocardial infarction; AUD, alcohol use disorder; Grimage, GrimAge epigenetic clock.

### 2.2. Data Source

All analytical data we used have been published. Genetic instruments of the HIV susceptibility came from FinnGen project (ID: finn‐b‐AB1_HIV), which involved 218,792 participants of European ancestry (357 cases and 218,435 controls). Genetic data for the outcome of AF were obtained from the biggest currently available meta‐analysis (ID: ebi‐a‐GCST006061), genome‐wide association study (GWAS). This analysis was based on 55,114 cases of European origin and 482,295 control cases, with details described in the corresponding publication [21].

To control for potential confounding factors, we selected genetic instruments for six key covariates. Smoking behavior data were obtained from the GSCAN project, which summarized data from approximately 1.2 million participants [22]. We selected the “cigarettes per day” phenotype to construct the genetic instrument. Genetic data for alcohol use disorder and obesity were sourced from the FinnGen R9 database, which included 15,715 cases and 361,562 controls for alcohol use disorder, and 21375 cases and 355786 controls for obesity, respectively. Statistics for MI were also sourced from the FinnGen R9 database, comprising 24,185 cases and 313,400 controls. Genetic associations for hypertension were derived from the Genetic Epidemiology Research on Adult Health and Aging (GERA) cohort, which included 28,391 cases and 28,246 controls [23]. We selected GrimAge, a DNA methylation‐based epigenetic clock, as the metric to quantify biological aging. Its genetic instruments were obtained from a GWAS analysis of age‐related DNA methylation biomarkers, which included 34,467 participants [24]. All participants were of European ancestry (see Table S1 for details).

### 2.3. Selection of Instrumental Variables

Based on the three assumptions above, we selected SNPs related to the exposures at the genome‐wide significance level (*p* < 5 × 10^−8^), a clumping window of 10,000 kb, with the linkage disequilibrium threshold of *r* ^2^ < 0.001. Given the limited number of SNPs available in the HIV dataset, we used a relaxed threshold of *p* < 5 × 10^−6^, which identified nine suitable SNPs. We then used the PhenoScanner V2 database to identify and exclude any instrumental variables that were related to potential confounders or the outcome. Then, SNPs exhibiting heterogeneity or outlier characteristics were removed. Finally, the *F*‐statistic was calculated as: *R* ^2^ = *β* ^2^ × 2 × MAF × (1 − MAF) and *F* = [(*N* − *k* − 1)/*k*] × [*R* ^2^/(1 − *R* ^2^)], where *N* represents the sample size, *β* is the effect size, *k* denotes the number of SNPs, and MAF is the minor allele frequency. Variants with an *F*‐statistic ≥ 10 were defined as strong genetic instruments [25]. More information about selected SNPs can be found in Table 1. During data harmonization, effect alleles were aligned between the exposure and outcome datasets, and palindromic SNPs with intermediate allele frequencies were excluded. Only SNPs present in both datasets were retained, with no imputation performed. Similarly, for the reverse MR analysis of AF on HIV infection, genetic variants for AF were identified using a genome‐wide significance threshold of *p* < 5 × 10^−8^ (see Table S2 for details).

**Table 1:** The selected IVs.

SNP	EA	OA	Chr	EAF	SNP‐HIV association			SNP‐AF association			*F*
					Beta	SE	*p*	Beta	SE	*p*	
rs13296311	A	G	9	0.125	–0.544	0.118	4.15e‐06	–0.023	0.016	0.144	15162
rs1373940	C	T	5	0.706	–0.403	0.084	1.70e‐06	0.004	0.008	0.606	15787
rs300163	A	C	2	0.295	0.380	0.083	4.97e‐06	0.016	0.008	0.052	14010
rs34628943	A	G	16	0.007	3.148	0.603	1.79e‐07	0.053	0.029	0.066	32408
rs56110037	T	C	14	0.487	–0.364	0.077	2.19e‐06	0.002	0.008	0.800	15493
rs78849743	T	C	17	0.045	0.948	0.196	1.31e‐06	–0.010	0.024	0.670	18424
rs79856406	G	T	2	0.102	0.592	0.129	4.75e‐06	0.022	0.015	0.142	15013
rs929671	C	A	22	0.693	–0.404	0.083	1.03e‐06	–0.010	0.008	0.213	16360

Abbreviations: Beta, beta coefficient; Chr, chromosome; EA, effect allele; EAF, effect allele frequency; OA, other allele; *p* value, probability value; SE, standard error; SNP, single nucleotide polymorphism; SNP‐AF association, single nucleotide polymorphism‐atrial fibrillation association; SNP‐HIV association, single nucleotide polymorphism‐human immunodeficiency virus association.

### 2.4. MR

We primarily used the random‐effects inverse‐variance weighted (IVW) method as the main analysis, as it provides the most statistically efficient effect estimates [26]. The weighted median (WM) method and MR‐Egger regression were used for supplementary validation. The MR‐Egger method can detect horizontal pleiotropy with fewer assumptions [27], and the WM method provides a valid causal estimate even when 50% of the SNPs are invalid [28].

We used Cochran′s *Q* statistic to assess the heterogeneity [26] and MR‐PRESSO method for global outlier detection. If any outlier SNP was identified, the causal effect was re‐estimated following the exclusion of any outlier SNPs detected [29]. The MR‐Egger was used to evaluate directional pleiotropy [30]. Finally, leave‐one‐out analysis was used to identify the high‐influence SNPs. Then we evaluated the changes in the effect estimates and statistical significance by sequentially eliminating these SNPs [31].

### 2.5. Statistical Analysis

Each analysis was performed using R software (Version 4.4.1), the TwoSampleMR (Version 0.6.22) [32], MendelianRandomization (Version 0.10.0) [33], and MR‐PRESSO (Version 1.0) packages were utilized within it [29].

## 3. Results

### 3.1. Causal Relationship Estimates for HIV on AF

Based on the above principles and criteria, we screened appropriate SNPs from the database and selected nine as instrumental variables, with rs17176972 excluded due to its palindromic sequence. All *F*‐statistics exceeded 10, confirming the strength and reliability of the selected instruments (see Table 1 for details). The eight selected SNPs collectively explained approximately 0.0836% of the variance in HIV infection, with individual *R* ^2^ values ranging from 0.0096% to 0.0125% (see Table S3 for details). Statistical power was estimated to be approximately 5% using the mRnd calculator.

We employed IVW as the main analytical method. As shown in Figure 2, the *p* value of the IVW method was 0.0106, and the OR value was 1.016 (95% CI: 1.0037–1.0285). The WM method yielded a consistent estimate (OR = 1.0175, 95% CI: 1.0010–1.0342, *p* = 0.0391). MR‐Egger (OR = 1.0168, 95% CI: 0.9949–1.0391, *p* = 0.1846) and MR‐PRESSO (OR = 1.0160, 95% CI: 1.0037–1.0285, *p* = 0.0377) also produced consistent estimates. These results suggest that genetically predicted HIV infection might be associated with an increased risk of AF. Scatter plots for the three methods are presented in Figure 3A. Cochran′s *Q* test showed that there was no heterogeneity (*Q* = 6.9965, *p* = 0.3212). The MR‐Egger intercept test (*p* = 0.9386) and the MR‐PRESSO global test (*p* = 0.545) indicated no directional pleiotropy or global outliers (Table 2). However, given the limited number of instruments, these tests may have reduced power to detect pleiotropy, which we acknowledge as a limitation in Section 4. Leave‐one‐out analysis (Figure 3B) showed that excluding single variants did not substantially change the overall estimate.

**Figure 2:**
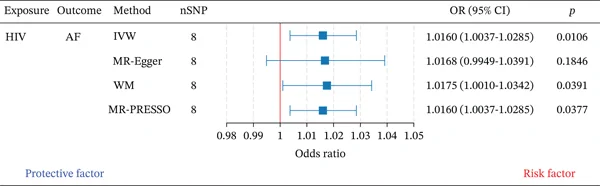
Forest plots of the causal effects of HIV infection on AF. HIV, human immunodeficiency virus; AF, atrial fibrillation; IVW, inverse‐variance weighted; WM, weighted median; MR‐PRESSO, Mendelian randomization pleiotropy residual sum and outlier; nSNP, number of single nucleotide polymorphisms; OR, odds ratio; CI, confidence interval.

**Figure 3:**
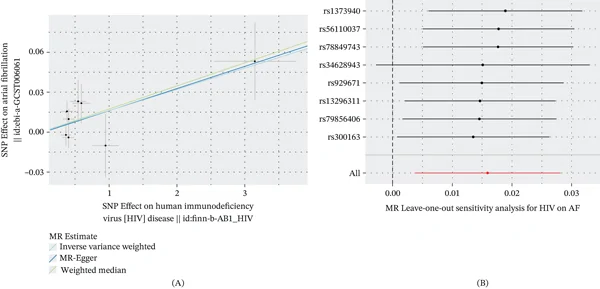
Scatter plot and leave‐one‐out plot of the causal effects of HIV infection on AF. (A) Scatter plot. (B) Leave‐one‐out plot. SNP, single nucleotide polymorphism; MR, Mendelian randomization.

**Table 2:** The results of MR‐Egger and Cochran′s *Q* test.

Exposure	Outcome	Cochran′s *Q* test		Egger intercept test		Global test
		*Q*statistic	*p*	Intercept	*p*	*p*
HIV	AF	6.9965	0.3212	−0.0005218	0.9386	0.545
AF	HIV	48.86002	0.8475	0.02872	0.2185	0.855

*Note:* Cochran′s *Q* test, Cochran′s *Q* heterogeneity test; Egger intercept test, Egger intercept test for pleiotropy; Global test, global pleiotropy test.

We also performed a reverse MR analysis. As shown in Figures 4 and 5A, there was no significant correlation across all methods. After conducting Cochran′s *Q* test, MR‐Egger intercept, and global test, sensitivity analysis proved that there was neither heterogeneity nor horizontal pleiotropy (Table 2). Results remain stable in the leave‐one‐out analysis (Figure 5B).

**Figure 4:**
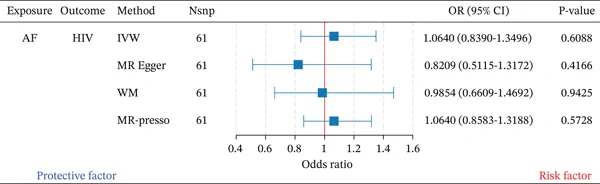
Forest plots of the causal effects of AF on HIV infection. AF, atrial fibrillation; HIV, human immunodeficiency virus; IVW, inverse‐variance weighted; MR‐Egger, Mendelian randomization Egger; WM, weighted median; MR‐PRESSO, Mendelian randomization pleiotropy residual sum and outlier; nSNP, number of single nucleotide polymorphisms; OR, odds ratio; CI, confidence interval.

**Figure 5:**
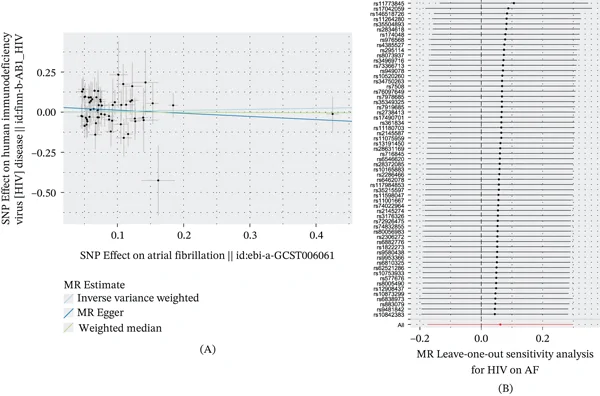
Scatter plot and leave‐one‐out plot of the causal effects of AF on HIV infection. (A) Scatter plot. (B) Leave‐one‐out plot. SNP, single nucleotide polymorphism; MR, Mendelian randomization.

### 3.2. Multivariable MR Analysis

To assess the independent effect of HIV on AF, we further conducted MVMR analyses. After separately adjusting for smoking, alcohol consumption, obesity, hypertension, MI, and GrimAge, the associations remained consistently significant, with odds ratios ranging from 1.014 (95% CI: 1.002–1.027) to 1.018 (95% CI: 1.005–1.033). Similar results were observed when these potential confounders were adjusted simultaneously (OR: 1.016, 95% CI: 1.002–1.031) (see Table 3 and Figure 6 for details). In models adjusting for each covariate separately, the conditional *F*‐statistic for HIV ranged from 4.79 to 15.55. When all six covariates were adjusted simultaneously, all conditional *F*‐statistics fell below 10 (range: 1.99–7.79) (see Table S4 for details). These findings indicate that some MVMR models are at risk of weak instrument bias, particularly the fully adjusted model, and their estimates should be interpreted with caution.

**Table 3:** The results of multivariable MR analysis.

Exposure	nSNP	OR (95% CI)	*p*
HIV + cigarette	34	1.0151 (1.0040, 1.0263)	0.009
HIV + AUD	15	1.0151 (1.0010, 1.0294)	0.036
HIV + obesity	36	1.0151 (1.0030, 1.0284)	0.016
HIV + hypertension	9	1.0212 (1.0010, 1.0418)	0.037
HIV + MI	51	1.0182 (1.0050, 1.0325)	0.009
HIV + Grimage	13	1.0141 (1.0020, 1.0274)	0.021
HIV + all confounders	90	1.0161 (1.0020, 1.0305)	0.028

*Note:* Cigarette, cigarettes per day; Grimage, GrimAge epigenetic clock.

Abbreviations: AUD, alcohol use disorder; CI, confidence interval; HIV, human immunodeficiency virus; MI, myocardial infarction; nSNP, number of single nucleotide polymorphisms; OR, odds ratio.

**Figure 6:**
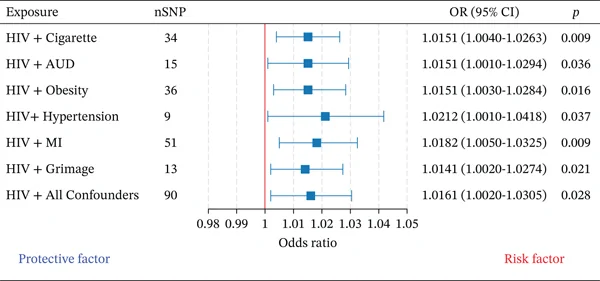
Forest plots of the results of MVMR. HIV, human immunodeficiency virus; nSNP, number of single nucleotide polymorphisms; OR, odds ratio; CI, confidence interval; MI, myocardial infarction; AUD, alcohol use disorder; Grimage, GrimAge epigenetic clock.

## 4. Discussion

To the best of our knowledge, this study was the first to investigate the causal association between HIV infection and AF risk. The univariate MR analysis revealed suggestive evidence of a positive causal effect of HIV on AF, with no evidence supporting a reverse causal relationship. This association remained robust after adjustment for potential confounders, including smoking, alcohol consumption, obesity, hypertension, MI, and GrimAge.

Advances in HIV prevention and treatment, including pre‐exposure prophylaxis (PrEP) and combination antiretroviral therapy (cART), have markedly reduced mortality and increased life expectancy among PLWH [5, 34]. For PLWH receiving standardized treatment and with good immune function recovery, their health outcomes have approached those of the general population [5]. However, this transition has accelerated the aging of PLWH. It is predicted that by 2030, nearly three‐quarters of PLWH will be over the age of 50 [35]. Consequently, age‐related diseases, particularly CVD, have become increasingly important determinants of long‐term health among PLWH. By 2030, the prevalence of CVD among PLWH will rise from 19% in 2010 to 78% [35]. This trend represents a substantial and growing disease burden. A previous analysis estimated that HIV accounted for approximately 0.92% of the global CVD burden in 2015, compared with 0.36% in 1990 [6]. Increasing epidemiological evidence suggests that PLWH have a significantly higher risk of developing AF. Accordingly, clarifying whether HIV infection causally contributes to AF holds important clinical and public health value for improving the long‐term prognosis of PLWH and alleviating the growing global burden of HIV‐related CVD.

Observational evidence regarding the relationship between HIV infection and AF remains limited and potentially confounded. Early cohort studies reported associations between markers of HIV disease severity and AF risk. For instance, a CD4+ T cell count lower than 200 cells/mm^3^ indicated a double risk of developing AF or atrial flutter [7]. This association was independent of cardiovascular risk factors [8]. Subsequent large‐scale epidemiological studies further confirmed this link, indicating that both baseline and newly diagnosed HIV infections are independent risk factors for incident AF [9]. This evidence is corroborated by a meta‐analysis of over 100,000 individuals, showing higher AF incidence in PLWH than in uninfected controls [10]. The observed association may partly reflect cardiovascular comorbidities or shared behavioral and metabolic risk factors, including smoking, alcohol consumption, obesity, and aging. At the same time, the treatment and complications of AF may affect the patient′s immune status, making it difficult to clearly determine the causal direction between HIV infection and AF. Therefore, our research uses genetic variations as instrumental variables. The random allocation characteristic of these variables largely avoids the aforementioned biases and makes up for the shortcomings of previous studies.

We speculated that the underlying mechanisms of the HIV‐related risk of AF may include the following aspects. First of all, inflammation has been proposed as a potential mechanism. Even if patients receive cART and achieve viral suppression, the residual viruses in their bodies and the translocation of intestinal microorganisms still lead to continuous immune activation, causing the body to be in a chronic low‐level inflammatory state [18]. The persistent inflammatory state may contribute to the occurrence and progression of AF [36]. Several circulating inflammatory biomarkers, which increase in PLWH [18, 37], have been independently proven to be associated with AF [38–40]. Secondly, research indicates that the trans‐activating protein of the HIV virus (Tat) has been reported to promote myocardial fibrosis by enhancing the transforming growth factor‐beta 1‐connective tissue growth factor (TGF‐*β*1‐CTGF) signaling cascade [41]. Certain antiretroviral drugs, particularly nucleoside reverse transcriptase inhibitors (NRTIs), may also directly damage myocardial function by inducing mitochondrial toxicity [42]. These direct viral effects, inflammatory response, and drug toxicity may contribute to a specific “HIV‐associated cardiomyopathy,” providing fertile ground for AF [43]. Thirdly, human immunodeficiency virus‐associated autonomic neuropathy (HIV‐AN) independently predicts long‐term adverse outcomes in PLWH, such as cardiovascular events [44]. A case–control study found a global reduction in heart rate variability (HRV) in the HIV‐positive group, which is a key marker of autonomic dysfunction [45]. Although mechanisms underlying HIV‐AN remain incompletely understood, we propose that HIV‐AN may promote AF through pathways similar to those characterized in diabetic autonomic neuropathy [46]. These proposed mechanisms are derived from previous literature and were not directly evaluated in the present study. Therefore, they should be regarded as hypotheses requiring experimental and clinical validation.

Previous evidence for HIV with CVD mainly focuses on hypertension and MI. A recent MR study by Zhu et al. [47] reported a causal association between HIV infection and elevated blood pressure. In addition, PLWH have been reported to have a 1.5‐ to 2‐fold higher risk of acute myocardial infarction (AMI) than the general population even in the setting of effective viral suppression [48, 49]. Because hypertension and MI are well‐known risk factors for AF, we additionally adjusted for these traits in our MR analysis and achieved consistent results with original analysis. These results suggested that the observed association between HIV and AF may not be fully explained by genetic liability to hypertension or MI and may involve additional pathways, such as virus‐mediated inflammation, immunity, and fibrotic pathways.

This article is the first to use MR to investigate the HIV‐AF link from a genetic perspective. MR leverages the approximately random allocation of genetic variants at conception to reduce susceptibility to confounding and reverse causation. The bidirectional MR analysis provided no evidence supporting a reverse effect of genetic liability to AF on HIV susceptibility. Multivariable MR further enabled adjustment for selected behavioral, metabolic, cardiovascular, and aging‐related traits. These complementary analyses strengthen the causal interpretation of the observed association. The data we used all come from large public databases such as OpenGWAS and FinnGen, with sufficient sample size, and the exposure and outcome data come from different populations, avoiding potential biases caused by sample overlap. At the same time, all data have passed relevant ethical reviews and have been publicly released for transparency and reproducibility of the research process.

This study fills some gaps in the field of causal research on HIV‐related cardiovascular complications. The effect size observed in our study was modest (OR = 1.016), suggesting that genetically predicted HIV susceptibility may confer a relatively small increase in AF risk. However, given the large and growing population of PLWH worldwide, even a small relative increase in risk may translate into a substantial number of excess AF cases at the population level. With the deepening of the cART treatment era, the challenges faced by HIV‐infected individuals have shifted from traditional opportunistic infections to a chronic comorbidity burden mainly consisting of CVDs. AF, as an important trigger for HF and stroke, has not been adequately evaluated in a systematic manner in the long‐term management of HIV. Our findings support further investigation of AF risk assessment and surveillance strategies in PLWH and also lay a research foundation for exploring intermediary mechanisms such as inflammation‐driven and immune aging.

Our study has some important limitations. Firstly, we used genetically predicted HIV infection status as the exposure variable, which captures genetic susceptibility to HIV rather than the full clinical course of HIV infection. The clinical phenotype of HIV infection covers multiple dimensions, including viral load, immune status, type and response of ART, and disease stage, which are not adequately captured by currently available HIV GWAS data. Therefore, the MR estimates should be interpreted as the causal effect of “genetically predicted HIV susceptibility” on AF risk, rather than the effect of clinical HIV progression or its treatment‐related complications. Future studies can utilize GWAS data with more detailed clinical phenotypes, such as HIV viral load, CD4+ T cell count, or ART treatment status, to further clarify the specific clinical dimensions that drive AF. Secondly, the number of genetic variations strongly related to HIV infection is limited, and the HIV GWAS sample size is relatively small. The eight instrumental variables collectively explained only 0.0836% of the variance, and the statistical power estimation based on the mRnd calculator was approximately 5%. These characteristics substantially limit the ability to detect a small causal effect and contribute to imprecision in the effect estimate. The small number of instruments also limits the power of pleiotropy‐sensitive analyses, such as MR‐Egger and MR‐PRESSO. Although the results of sensitivity analyses such as WM, MR‐Egger, MR‐PRESSO and the leave‐one‐out method are highly consistent in direction, given the limited number of instrumental variables and the fact that residual pleiotropy cannot be fully excluded, the conclusions of this study should be regarded as suggestive evidence rather than definitive conclusions. Thirdly, all GWAS datasets were derived from individuals of European ancestry. Genetic background, HIV epidemiology, treatment patterns, and cardiovascular risk profiles vary substantially across populations, which may limit the generalizability of our findings to non‐European populations. Therefore, our results should be interpreted within European‐ancestry populations, and future studies are needed to validate these findings in diverse racial and regional populations. Finally, the present study did not directly explore the biological mechanism. Recent studies have begun to integrate single‐cell technology with the causal inference framework to address such mechanistic questions [50, 51]. Future research that combines single‐cell transcriptomics analysis with the mediation analysis framework based on MR could help cell type–specific effects and pathway‐level mediators behind the relationship between HIV and AF, thereby converting the statistical association established in this study into a causal model with clear biological mechanisms. Additionally, using more homogeneous clinical cohorts, more advanced MR methods, or larger scale GWAS data will also help to further validate and refine the findings of this study.

## 5. Conclusion

Our study provides suggestive evidence for a potential causal association between HIV infection and AF risk in populations of European ancestry, and the association was independent of potential confounders. Future studies are needed to confirm our findings in diverse populations and explore the underlying mechanisms.

**Nomenclature**
ACE: angiotensin‐converting enzyme
AF: atrial fibrillation
AICM: arrhythmia‐induced cardiomyopathy
AIDS: acquired immunodeficiency syndrome
AMI: acute myocardial infarction
ATP: adenosine triphosphate
cART: combination antiretroviral therapy
CRP: C‐reactive protein
EAT: epicardial adipose tissue
GERA: Genetic Epidemiology Research on Adult Health and Aging
GWAS: genome‐wide association study
HARRT: highly active antiretroviral therapy
HIV: human immunodeficiency virus
HIV‐AN: HIV‐associated autonomic neuropathy
HRV: heart rate variability
IL‐6: interleukin‐6
IVW: inverse‐variance weighted
MR: Mendelian randomization
mtDNA: mitochondrial DNA
MVMR: multivariate Mendelian randomization
NPs: natriuretic peptides
NRTIs: nucleoside reverse transcriptase inhibitors
PLWH: people living with HIV
RAAS: renin–angiotensin–aldosterone system
SERCA: sarcoplasmic reticulum calcium pump
SNPs: single nucleotide polymorphisms
Tat: trans‐activating protein of the HIV virus
TGF‐*β*
: transforming growth factor‐beta
TGF‐*β*1‐CTGF: transforming growth factor‐beta 1‐connective tissue growth factor
TNF‐*α*
: tumor necrosis factor‐alpha
UVMR: univariable Mendelian randomization
VDR: vitamin D receptor
WM: weighted median

## Author Contributions

Y.W. and X.C. were responsible for selecting topics and writing the manuscript text. W.P. collected the data and calculated the results. H.L., R.H., and M.Z. prepared the figures and tables. X.C., T.J., and X.L. reviewed the manuscript and supervised the project. Y.W. and W.P. contributed equally to this work and share the first authorship; Y.W. is listed first. X.C. is the first corresponding author. T.J. and X.L. are cocorresponding authors.

## Funding

This study is funded by the National Natural Science Foundation of China (10.13039/501100001809, 82400303).

## Disclosure

All authors read and approved the final manuscript.

## Ethics Statement

The authors have nothing to report.

## Consent

The authors have nothing to report.

## Conflicts of Interest

The authors declare no conflicts of interest.

## Supporting information


**Supporting Information** Additional supporting information can be found online in the Supporting Information section. Table S1: The detailed information on GWAS data source. Table S2: The selected IVs of AF on HIV. Table S3: *R* ^2^ of individual instrumental variables for HIV infection. Table S4: Conditional *F*‐statistics for each multivariable MR model.

## Data Availability

The GWAS summary statistics used in this study are publicly available from the IEU OpenGWAS database (https://gwas.mrcieu.ac.uk/) and the FinnGen study (https://www.finngen.fi/en). Accession numbers and dataset identifiers for all exposures and outcomes are provided in Section 2 and Supporting Information. Further inquiries can be directed to the corresponding authors. The R code used for all analyses is available from the corresponding authors upon reasonable request.

## References

[1] January C. T. , Wann L. S. , Alpert J. S. , Calkins H. , Cigarroa J. E. , Cleveland J. C. , Conti J. B. , Ellinor P. T. , Ezekowitz M. D. , Field M. E. , and Murray K. T. , 2014 AHA/ACC/HRS Guideline for the Management of Patients With Atrial Fibrillation: A Report of the American College of Cardiology/American Heart Association Task Force on Practice Guidelines and the Heart Rhythm Society, Circulation. (2014) 64, no. 21, e199–e267, 10.1016/j.jacc.2014.03.022, 24685669. 24685669

[2] Joglar J. A. , Chung M. K. , Armbruster A. L. , Benjamin E. J. , Chyou J. Y. , Cronin E. M. , Deswal A. , Eckhardt L. L. , Goldberger Z. D. , Gopinathannair R. , Gorenek B. , Hess P. L. , Hlatky M. , Hogan G. , Ibeh C. , Indik J. H. , Kido K. , Kusumoto F. , Link M. S. , Linta K. T. , Marcus G. M. , McCarthy P. , Patel N. , Patton K. K. , Perez M. V. , Piccini J. P. , Russo A. M. , Sanders P. , Streur M. M. , Thomas K. L. , Times S. , Tisdale J. E. , Valente A. M. , van Wagoner D. , and Peer Review Committee Members , 2023 ACC/AHA/ACCP/HRS Guideline for the Diagnosis and Management of Atrial Fibrillation: A Report of the American College of Cardiology/American Heart Association Joint Committee on Clinical Practice Guidelines, Circulation. (2024) 149, no. 1, e1–e156, 10.1161/CIR.0000000000001193, 38033089. PMC11095842 38033089

[3] Chyou J. Y. , Barkoudah E. , Dukes J. W. , Goldstein L. B. , Joglar J. A. , Lee A. M. , Lubitz S. A. , Marill K. A. , Sneed K. B. , Streur M. M. , Wong G. C. , Gopinathannair R. , on behalf of the American Heart Association Acute Cardiac Care and General Cardiology Committee , Electrocardiography and Arrhythmias Committee, and Clinical Pharmacology Committee of the Council on Clinical Cardiology , Council on Cardiovascular Surgery and Anesthesia , Council on Cardiopulmonary, Critical Care, Perioperative and Resuscitation , and Council on Cardiovascular and Stroke Nursing; and Stroke Council , Atrial Fibrillation Occurring During Acute Hospitalization: A Scientific Statement From the American Heart Association, Circulation. (2023) 147, no. 15, e676–e698, 10.1161/CIR.0000000000001133, 36912134. 36912134

[4] 2024 Global AIDS Report — The Urgency of Now: AIDS at a Crossroads. [https://www.unaids.org/en/resources/documents/2024/global-aids-update-2024, ]. 16 October 2025.

[5] Trickey A. , Sabin C. A. , Burkholder G. , Crane H. , d′Arminio Monforte A. , Egger M. , Gill M. J. , Grabar S. , Guest J. L. , Jarrin I. , Lampe F. C. , Obel N. , Reyes J. M. , Stephan C. , Sterling T. R. , Teira R. , Touloumi G. , Wasmuth J. C. , Wit F. , Wittkop L. , Zangerle R. , Silverberg M. J. , Justice A. , and Sterne J. A. C. , Life Expectancy After 2015 of Adults With HIV on Long-Term Antiretroviral Therapy in Europe and North America: A Collaborative Analysis of Cohort Studies, Lancet HIV. (2023) 10, no. 5, e295–e307, 10.1016/S2352-3018(23)00028-0, 36958365. PMC10288029 36958365

[6] Feinstein M. J. , Hsue P. Y. , Benjamin L. A. , Bloomfield G. S. , Currier J. S. , Freiberg M. S. , Grinspoon S. K. , Levin J. , Longenecker C. T. , Post W. S. , and On behalf of the American Heart Association Prevention Science Committee of the Council on Epidemiology and Prevention and Council on Cardiovascular and Stroke Nursing; Council on Clinical Cardiology; and Stroke Council , Characteristics, Prevention, and Management of Cardiovascular Disease in People Living With HIV: A Scientific Statement From the American Heart Association, Circulation. (2019) 140, no. 2, e98–e124, 10.1161/CIR.0000000000000695, 31154814. PMC7993364 31154814

[7] Sanders J. M. , Steverson A. B. , Pawlowski A. E. , Schneider D. , Achenbach C. J. , Lloyd-Jones D. M. , and Feinstein M. J. , Atrial Arrhythmia Prevalence and Characteristics for Human Immunodeficiency Virus-Infected Persons and Matched Uninfected Controls, PLoS One. (2018) 13, no. 3, e0194754, 10.1371/journal.pone.0194754, 29558525. PMC5860783 29558525

[8] Hsu J. C. , Li Y. , Marcus G. M. , Hsue P. Y. , Scherzer R. , Grunfeld C. , and Shlipak M. G. , Atrial Fibrillation and Atrial Flutter in Human Immunodeficiency Virus-Infected Persons: Incidence, Risk Factors, and Association With Markers of HIV Disease Severity, Journal of the American College of Cardiology. (2013) 61, no. 22, 2288–2295, 10.1016/j.jacc.2013.03.022, 23563125. 23563125

[9] Sardana M. , Hsue P. Y. , Tseng Z. H. , Vittinghoff E. , Nah G. , Dewland T. A. , and Marcus G. M. , Human Immunodeficiency Virus Infection and Incident Atrial Fibrillation, Journal of the American College of Cardiology. (2019) 74, no. 11, 1512–1514, 10.1016/j.jacc.2019.07.027. 31514955

[10] Park D. Y. , An S. , Romero M. E. , Kaur A. , Ravi V. , Huang H. D. , and Vij A. , Incidence and Risk Factors of Atrial Fibrillation and Atrial Arrhythmias in People Living With HIV: A Systematic Review and Meta-Analysis, Journal of Interventional Cardiac Electrophysiology. (2022) 65, no. 1, 183–191, 10.1007/s10840-022-01233-w. 35610524

[11] Voskoboinik A. , Prabhu S. , Ling L. H. , Kalman J. M. , and Kistler P. M. , Alcohol and Atrial Fibrillation: A Sobering Review, Journal of the American College of Cardiology. (2016) 68, no. 23, 2567–2576, 10.1016/j.jacc.2016.08.074. 27931615

[12] Aune D. , Schlesinger S. , Norat T. , and Riboli E. , Tobacco Smoking and the Risk of Atrial Fibrillation: A Systematic Review and Meta-Analysis of Prospective Studies, European Journal of Preventive Cardiology. (2018) 25, no. 13, 1437–1451, 10.1177/2047487318780435. 29996680

[13] Tesoriero J. M. , Gieryic S. M. , Carrascal A. , and Lavigne H. E. , Smoking Among HIV Positive New Yorkers: Prevalence, Frequency, and Opportunities for Cessation, AIDS and Behavior. (2010) 14, no. 4, 824–835, 10.1007/s10461-008-9449-2. 18777131

[14] Sax P. E. , Erlandson K. M. , Lake J. E. , McComsey G. A. , Orkin C. , Esser S. , Brown T. T. , Rockstroh J. K. , Wei X. , Carter C. C. , and Zhong L. , Weight Gain Following Initiation of Antiretroviral Therapy: Risk Factors in Randomized Comparative Clinical Trials, Clinical Infectious Diseases. (2020) 71, no. 6, 1379–1389, 10.1093/cid/ciz999, 31606734. PMC7486849 31606734

[15] Morseth B. , Geelhoed B. , Linneberg A. , Johansson L. , Kuulasmaa K. , Salomaa V. , Iacoviello L. , Costanzo S. , Soderberg S. , Niiranen T. J. , and Vishram-Nielsen J. K. , Age-Specific Atrial Fibrillation Incidence, Attributable Risk Factors and Risk of Stroke and Mortality: Results From the MORGAM Consortium, Heart. (2021) 8, no. 2, 10.1136/openhrt-2021-001624, 34341095. PMC8330568 34341095

[16] Dzeshka M. S. , Shantsila A. , Shantsila E. , and Lip G. Y. H. , Atrial Fibrillation and Hypertension, Hypertension. (2017) 70, no. 5, 854–861, 10.1161/HYPERTENSIONAHA.117.08934. 28893897

[17] Gupta V. , Munjal J. S. , Jhajj P. , Jhajj S. , and Jain R. , Obesity and Atrial Fibrillation: A Narrative Review, Cureus. (2022) 14, no. 11, e31205, 10.7759/cureus.31205. PMC9728938 36505155

[18] Deeks S. G. , HIV Infection, Inflammation, Immunosenescence, and Aging, Annual Review of Medicine. (2011) 62, 141–155, 10.1146/annurev-med-042909-093756. PMC3759035 21090961

[19] Emdin C. A. , Khera A. V. , and Kathiresan S. , Mendelian Randomization, JAMA. (2017) 318, no. 19, 1925–1926, 10.1001/jama.2017.17219. 29164242

[20] Sanderson E. , Multivariable Mendelian Randomization and Mediation, Cold Spring Harbor Perspectives in Medicine. (2021) 11, no. 2, 10.1101/cshperspect.a038984. PMC7849347 32341063

[21] Roselli C. , Chaffin M. D. , Weng L. C. , Aeschbacher S. , Ahlberg G. , Albert C. M. , Almgren P. , Alonso A. , Anderson C. D. , Aragam K. G. , Arking D. E. , Barnard J. , Bartz T. M. , Benjamin E. J. , Bihlmeyer N. A. , Bis J. C. , Bloom H. L. , Boerwinkle E. , Bottinger E. B. , Brody J. A. , Calkins H. , Campbell A. , Cappola T. P. , Carlquist J. , Chasman D. I. , Chen L. Y. , Chen Y. D. I. , Choi E. K. , Choi S. H. , Christophersen I. E. , Chung M. K. , Cole J. W. , Conen D. , Cook J. , Crijns H. J. , Cutler M. J. , Damrauer S. M. , Daniels B. R. , Darbar D. , Delgado G. , Denny J. C. , Dichgans M. , Dörr M. , Dudink E. A. , Dudley S. C. , Esa N. , Esko T. , Eskola M. , Fatkin D. , Felix S. B. , Ford I. , Franco O. H. , Geelhoed B. , Grewal R. P. , Gudnason V. , Guo X. , Gupta N. , Gustafsson S. , Gutmann R. , Hamsten A. , Harris T. B. , Hayward C. , Heckbert S. R. , Hernesniemi J. , Hocking L. J. , Hofman A. , Horimoto A. R. V. R. , Huang J. , Huang P. L. , Huffman J. , Ingelsson E. , Ipek E. G. , Ito K. , Jimenez-Conde J. , Johnson R. , Jukema J. W. , Kääb S. , Kähönen M. , Kamatani Y. , Kane J. P. , Kastrati A. , Kathiresan S. , Katschnig-Winter P. , Kavousi M. , Kessler T. , Kietselaer B. L. , Kirchhof P. , Kleber M. E. , Knight S. , Krieger J. E. , Kubo M. , Launer L. J. , Laurikka J. , Lehtimäki T. , Leineweber K. , Lemaitre R. N. , Li M. , Lim H. E. , Lin H. J. , Lin H. , Lind L. , Lindgren C. M. , Lokki M. L. , London B. , Loos R. J. F. , Low S. K. , Lu Y. , Lyytikäinen L. P. , Macfarlane P. W. , Magnusson P. K. , Mahajan A. , Malik R. , Mansur A. J. , Marcus G. M. , Margolin L. , Margulies K. B. , März W. , McManus D. D. , Melander O. , Mohanty S. , Montgomery J. A. , Morley M. P. , Morris A. P. , Müller-Nurasyid M. , Natale A. , Nazarian S. , Neumann B. , Newton-Cheh C. , Niemeijer M. N. , Nikus K. , Nilsson P. , Noordam R. , Oellers H. , Olesen M. S. , Orho-Melander M. , Padmanabhan S. , Pak H. N. , Paré G. , Pedersen N. L. , Pera J. , Pereira A. , Porteous D. , Psaty B. M. , Pulit S. L. , Pullinger C. R. , Rader D. J. , Refsgaard L. , Ribasés M. , Ridker P. M. , Rienstra M. , Risch L. , Roden D. M. , Rosand J. , Rosenberg M. A. , Rost N. , Rotter J. I. , Saba S. , Sandhu R. K. , Schnabel R. B. , Schramm K. , Schunkert H. , Schurman C. , Scott S. A. , Seppälä I. , Shaffer C. , Shah S. , Shalaby A. A. , Shim J. , Shoemaker M. B. , Siland J. E. , Sinisalo J. , Sinner M. F. , Slowik A. , Smith A. V. , Smith B. H. , Smith J. G. , Smith J. D. , Smith N. L. , Soliman E. Z. , Sotoodehnia N. , Stricker B. H. , Sun A. , Sun H. , Svendsen J. H. , Tanaka T. , Tanriverdi K. , Taylor K. D. , Teder-Laving M. , Teumer A. , Thériault S. , Trompet S. , Tucker N. R. , Tveit A. , Uitterlinden A. G. , van der Harst P. , van Gelder I. C. , van Wagoner D. R. , Verweij N. , Vlachopoulou E. , Völker U. , Wang B. , Weeke P. E. , Weijs B. , Weiss R. , Weiss S. , Wells Q. S. , Wiggins K. L. , Wong J. A. , Woo D. , Worrall B. B. , Yang P. S. , Yao J. , Yoneda Z. T. , Zeller T. , Zeng L. , Lubitz S. A. , Lunetta K. L. , and Ellinor P. T. , Multi-Ethnic Genome-Wide Association Study for Atrial Fibrillation, Nature Genetics. (2018) 50, no. 9, 1225–1233, 10.1038/s41588-018-0133-9, 29892015. PMC6136836 29892015

[22] Liu M. , Jiang Y. , Wedow R. , Li Y. , Brazel D. M. , Chen F. , Datta G. , Davila-Velderrain J. , McGuire D. , Tian C. , and Zhan X. , Association Studies of Up to 1.2 Million Individuals Yield New Insights Into the Genetic Etiology of Tobacco and Alcohol Use, Nature Genetics. (2019) 51, no. 2, 237–244, 10.1038/s41588-018-0307-5, 30643251. PMC6358542 30643251

[23] Guindo-Martinez M. , Amela R. , Bonas-Guarch S. , Puiggros M. , Salvoro C. , Miguel-Escalada I. , Carey C. E. , Cole J. B. , Rueger S. , Atkinson E. , and Leong A. , The Impact of Non-Additive Genetic Associations on Age-Related Complex Diseases, Nature Communications. (2021) 12, no. 1, 10.1038/s41467-021-21952-4. PMC8065056 33893285

[24] McCartney D. L. , Min J. L. , Richmond R. C. , Lu A. T. , Sobczyk M. K. , Davies G. , Broer L. , Guo X. , Jeong A. , Jung J. , Kasela S. , Katrinli S. , Kuo P. L. , Matias-Garcia P. R. , Mishra P. P. , Nygaard M. , Palviainen T. , Patki A. , Raffield L. M. , Ratliff S. M. , Richardson T. G. , Robinson O. , Soerensen M. , Sun D. , Tsai P. C. , van der Zee M. D. , Walker R. M. , Wang X. , Wang Y. , Xia R. , Xu Z. , Yao J. , Zhao W. , Correa A. , Boerwinkle E. , Dugué P. A. , Durda P. , Elliott H. R. , Gieger C. , The Genetics of DNA Methylation Consortium , de Geus E. J. C. , Harris S. E. , Hemani G. , Imboden M. , Kähönen M. , Kardia S. L. R. , Kresovich J. K. , Li S. , Lunetta K. L. , Mangino M. , Mason D. , McIntosh A. M. , Mengel-From J. , Moore A. Z. , Murabito J. M. , NHLBI Trans-Omics for Precision Medicine (TOPMed) Consortium , Ollikainen M. , Pankow J. S. , Pedersen N. L. , Peters A. , Polidoro S. , Porteous D. J. , Raitakari O. , Rich S. S. , Sandler D. P. , Sillanpää E. , Smith A. K. , Southey M. C. , Strauch K. , Tiwari H. , Tanaka T. , Tillin T. , Uitterlinden A. G. , van den Berg D. J. , van Dongen J. , Wilson J. G. , Wright J. , Yet I. , Arnett D. , Bandinelli S. , Bell J. T. , Binder A. M. , Boomsma D. I. , Chen W. , Christensen K. , Conneely K. N. , Elliott P. , Ferrucci L. , Fornage M. , Hägg S. , Hayward C. , Irvin M. , Kaprio J. , Lawlor D. A. , Lehtimäki T. , Lohoff F. W. , Milani L. , Milne R. L. , Probst-Hensch N. , Reiner A. P. , Ritz B. , Rotter J. I. , Smith J. A. , Taylor J. A. , van Meurs J. B. J. , Vineis P. , Waldenberger M. , Deary I. J. , Relton C. L. , Horvath S. , and Marioni R. E. , Genome-Wide Association Studies Identify 137 Genetic Loci for DNA Methylation Biomarkers of Aging, Genome Biology. (2021) 22, no. 1, 10.1186/s13059-021-02398-9, 34187551. PMC8243879 34187551

[25] Burgess S. , Thompson S. G. , and Collaboration CCG , Avoiding Bias From Weak Instruments in Mendelian Randomization Studies, International Journal of Epidemiology. (2011) 40, no. 3, 755–764, 10.1093/ije/dyr036. 21414999

[26] Burgess S. , Dudbridge F. , and Thompson S. G. , Combining Information on Multiple Instrumental Variables in Mendelian Randomization: Comparison of Allele Score and Summarized Data Methods, Statistics in Medicine. (2016) 35, no. 11, 1880–1906, 10.1002/sim.6835. PMC4832315 26661904

[27] Burgess S. and Thompson S. G. , Interpreting Findings From Mendelian Randomization Using the MR-Egger Method, European Journal of Epidemiology. (2017) 32, no. 5, 377–389, 10.1007/s10654-017-0255-x. PMC5506233 28527048

[28] Bowden J. , Davey Smith G. , Haycock P. C. , and Burgess S. , Consistent Estimation in Mendelian Randomization With Some Invalid Instruments Using a Weighted Median Estimator, Genetic Epidemiology. (2016) 40, no. 4, 304–314, 10.1002/gepi.21965. PMC4849733 27061298

[29] Verbanck M. , Chen C. Y. , Neale B. , and Do R. , Detection of Widespread Horizontal Pleiotropy in Causal Relationships Inferred From Mendelian Randomization Between Complex Traits and Diseases, Nature Genetics. (2018) 50, no. 5, 693–698, 10.1038/s41588-018-0099-7. PMC6083837 29686387

[30] Bowden J. , Davey Smith G. , and Burgess S. , Mendelian Randomization With Invalid Instruments: Effect Estimation and Bias Detection Through Egger Regression, International Journal of Epidemiology. (2015) 44, no. 2, 512–525, 10.1093/ije/dyv080. PMC4469799 26050253

[31] Sanderson E. , Glymour M. M. , Holmes M. V. , Kang H. , Morrison J. , Munafo M. R. , Palmer T. , Schooling C. M. , Wallace C. , Zhao Q. , and Davey Smith G. , Mendelian Randomization, Nature Reviews Methods Primers. (2022) 2, 10.1038/s43586-021-00092-5, 37325194. PMC7614635 37325194

[32] Hemani G. , Zheng J. , Elsworth B. , Wade K. H. , Haberland V. , Baird D. , Laurin C. , Burgess S. , Bowden J. , Langdon R. , Tan V. Y. , Yarmolinsky J. , Shihab H. A. , Timpson N. J. , Evans D. M. , Relton C. , Martin R. M. , Davey Smith G. , Gaunt T. R. , and Haycock P. C. , The MR-Base Platform Supports Systematic Causal Inference Across the Human Phenome, Elife. (2018) 7, 10.7554/eLife.34408, 29846171. PMC5976434 29846171

[33] Yavorska O. O. and Burgess S. , MendelianRandomization: An R Package for Performing Mendelian Randomization Analyses Using Summarized Data, International Journal of Epidemiology. (2017) 46, no. 6, 1734–1739, 10.1093/ije/dyx034. PMC5510723 28398548

[34] Grant R. M. , Lama J. R. , Anderson P. L. , McMahan V. , Liu A. Y. , Vargas L. , Goicochea P. , Casapia M. , Guanira-Carranza J. V. , Ramirez-Cardich M. E. , and Montoya-Herrera O. , Preexposure Chemoprophylaxis for HIV Prevention in Men Who Have Sex With Men, New England Journal of Medicine. (2010) 363, no. 27, 2587–2599, 10.1056/NEJMoa1011205, 21091279. PMC3079639 21091279

[35] Smit M. , Brinkman K. , Geerlings S. , Smit C. , Thyagarajan K. , Sighem A. , de Wolf F. , and Hallett T. B. , Cohort Ao: Future Challenges for Clinical Care of an Ageing Population Infected With HIV: A Modelling Study, Lancet Infectious Diseases. (2015) 15, no. 7, 810–818, 10.1016/S1473-3099(15)00056-0. PMC4528076 26070969

[36] Hu Y. F. , Chen Y. J. , Lin Y. J. , and Chen S. A. , Inflammation and the Pathogenesis of Atrial Fibrillation, Nature Reviews Cardiology. (2015) 12, no. 4, 230–243, 10.1038/nrcardio.2015.2. 25622848

[37] Neuhaus J. , Jacobs D. R. , Baker J. V. , Calmy A. , Duprez D. , La Rosa A. , Kuller L. H. , Pett S. L. , Ristola M. , Ross M. J. , and Shlipak M. G. , Markers of Inflammation, Coagulation, and Renal Function Are Elevated in Adults With HIV Infection, Journal of Infectious Diseases. (2010) 201, no. 12, 1788–1795, 10.1086/652749, 20446848. PMC2872049 20446848

[38] Aviles R. J. , Martin D. O. , Apperson-Hansen C. , Houghtaling P. L. , Rautaharju P. , Kronmal R. A. , Tracy R. P. , Van Wagoner D. R. , Psaty B. M. , Lauer M. S. , and Chung M. K. , Inflammation as a Risk Factor for Atrial Fibrillation, Circulation. (2003) 108, no. 24, 3006–3010, 10.1161/01.CIR.0000103131.70301.4F. 14623805

[39] Liao J. , Zhang S. , Yang S. , Lu Y. , Lu K. , Wu Y. , Wu Q. , Zhao N. , Dong Q. , Chen L. , and du Y. , Interleukin-6-Mediated-Ca(2+) Handling Abnormalities Contributes to Atrial Fibrillation in Sterile Pericarditis Rats, Frontiers in Immunology. (2021) 12, 758157, 10.3389/fimmu.2021.758157, 34975847. PMC8716408 34975847

[40] Lee S. H. , Chen Y. C. , Chen Y. J. , Chang S. L. , Tai C. T. , Wongcharoen W. , Yeh H. I. , Lin C. I. , and Chen S. A. , Tumor Necrosis Factor-Alpha Alters Calcium Handling and Increases Arrhythmogenesis of Pulmonary Vein Cardiomyocytes, Life Sciences. (2007) 80, no. 19, 1806–1815, 10.1016/j.lfs.2007.02.029. 17383682

[41] Jiang Y. , Chai L. , Wang H. , Shen X. , Fasae M. B. , Jiao J. , Yu Y. , Ju J. , Liu B. , and Bai Y. , HIV Tat Protein Induces Myocardial Fibrosis Through TGF-Beta1-CTGF Signaling Cascade: A Potential Mechanism of HIV Infection-Related Cardiac Manifestations, Cardiovascular Toxicology. (2021) 21, no. 12, 965–972, 10.1007/s12012-021-09687-6. 34519946

[42] Crain M. J. , Chernoff M. C. , Oleske J. M. , Brogly S. B. , Malee K. M. , Borum P. R. , Meyer W. A. , Mitchell W. G. , Moye J. H. , Ford-Chatterton H. M. , and Van Dyke R. B. , Possible Mitochondrial Dysfunction and Its Association With Antiretroviral Therapy Use in Children Perinatally Infected With HIV, Journal of Infectious Diseases. (2010) 202, no. 2, 291–301, 10.1086/653497, 20533872. PMC2913975 20533872

[43] Remick J. , Georgiopoulou V. , Marti C. , Ofotokun I. , Kalogeropoulos A. , Lewis W. , and Butler J. , Heart Failure in Patients With Human Immunodeficiency Virus Infection: Epidemiology, Pathophysiology, Treatment, and Future Research, Circulation. (2014) 129, no. 17, 1781–1789, 10.1161/CIRCULATIONAHA.113.004574. PMC4006343 24778120

[44] Kwon P. M. , Lawrence S. , Figueroa A. , and Robinson-Papp J. , Autonomic Neuropathy as a Predictor of Morbidity and Mortality in People Living With HIV: A Retrospective, Longitudinal Cohort Study, Neurology: Clinical Practice. (2023) 13, no. 3, e200141, 10.1212/CPJ.0000000000200141. PMC10092299 37066108

[45] Wongcharoen W. , Khienprasit K. , Phrommintikul A. , Sukonthasarn A. , and Chattipakorn N. , Heart Rate Variability and Heart Rate Turbulence in HIV-Infected Patients Receiving Combination Antiretroviral Therapy, Annals of Noninvasive Electrocardiology. (2013) 18, no. 5, 450–456, 10.1111/anec.12064. PMC6932603 24047489

[46] Wang A. , Green J. B. , Halperin J. L. , and Piccini J. P. , Atrial Fibrillation and Diabetes Mellitus: JACC Review Topic of the Week, Journal of the American College of Cardiology. (2019) 74, no. 8, 1107–1115, 10.1016/j.jacc.2019.07.020. 31439220

[47] Zhu R. W. , Guo H. Y. , Niu L. N. , Deng M. , Li X. F. , and Jing L. , HIV and Risk of Hypertension: A Two-Sample Mendelian Randomization Study, BMC Infectious Diseases. (2024) 24, no. 1, 10.1186/s12879-024-10182-w. PMC11585946 39580426

[48] Shah A. S. V. , Stelzle D. , Lee K. K. , Beck E. J. , Alam S. , Clifford S. , Longenecker C. T. , Strachan F. , Bagchi S. , Whiteley W. , Rajagopalan S. , Kottilil S. , Nair H. , Newby D. E. , McAllister D. A. , and Mills N. L. , Global Burden of Atherosclerotic Cardiovascular Disease in People Living With HIV: Systematic Review and Meta-Analysis, Circulation. (2018) 138, no. 11, 1100–1112, 10.1161/CIRCULATIONAHA.117.033369, 29967196. PMC6221183 29967196

[49] Freiberg M. S. , Chang C. C. , Kuller L. H. , Skanderson M. , Lowy E. , Kraemer K. L. , Butt A. A. , Bidwell Goetz M. , Leaf D. , Oursler K. A. , Rimland D. , Rodriguez Barradas M. , Brown S. , Gibert C. , McGinnis K. , Crothers K. , Sico J. , Crane H. , Warner A. , Gottlieb S. , Gottdiener J. , Tracy R. P. , Budoff M. , Watson C. , Armah K. A. , Doebler D. , Bryant K. , and Justice A. C. , HIV Infection and the Risk of Acute Myocardial Infarction, JAMA Internal Medicine. (2013) 173, no. 8, 614–622, 10.1001/jamainternmed.2013.3728, 23459863. PMC4766798 23459863

[50] Li K. , Deng J. , Zhang C. , Lai G. , Xie B. , and Zhong X. , Gut Microbiome Dysbiosis in Men Who Have Sex With Men Increases HIV Infection Risk Through Immunity Homeostasis Alteration, Frontiers in Cellular and Infection Microbiology. (2023) 13, 1260068, 10.3389/fcimb.2023.1260068. PMC10687210 38035339

[51] Liu H. , Lai G. , Shi G. , and Zhong X. , The Influencing Factors of HIV-Preventive Behavior Based on Health Belief Model Among HIV-Negative MSMs in Western China: A Structural Equation Modeling Analysis, International Journal of Environmental Research and Public Health. (2022) 19, no. 16, 10.3390/ijerph191610185. PMC9407807 36011822

